# Characterization of German SF_6_ Emissions

**DOI:** 10.1021/acsestair.5c00234

**Published:** 2025-11-06

**Authors:** Katharina Meixner, Thomas Wagenhäuser, Tanja J. Schuck, Sascha Alber, Alistair J. Manning, Alison L. Redington, Kieran M. Stanley, Simon O’Doherty, Dickon Young, Joseph Pitt, Angelina Wenger, Arnoud Frumau, Ann R. Stavert, Christopher Rennick, Martin K. Vollmer, Michela Maione, Jgor Arduini, Chris R. Lunder, Cedric Couret, Armin Jordan, Xochilt Gutiérrez Gutiérrez, Dagmar Kubistin, Jennifer Müller-Williams, Matthias Lindauer, Martin Vojta, Andreas Stohl, Andreas Engel

**Affiliations:** † Institute of Atmospheric and Environmental Sciences, Goethe University, Frankfurt am Main 60438, Germany; ‡ Institute for Energy and Climate Research (IEK-7: Stratosphere), Forschungszentrum Jülich GmbH, Jülich 52425, Germany; § 11365 Met Office, Hadley Centre, Exeter EX1 3PB, U.K.; ∥ School of Chemistry, 1980 University of Bristol, Bristol BS8 1QU, U.K.; ⊥ The Netherlands Organisation for Applied Scientific Research TNO, Den Haag 2509 JE, Netherlands; # CSIRO Environment, Black Mountain, Canberra ACT 2601, Australia; ¶ 9917 National Physical Laboratory, Teddington TW11 0LW, U.K.; ∇ Laboratory for Air Pollution and Environmental Technology, Empa, Swiss Federal Laboratories for Materials Science and Technology, Dübendorf 8600, Switzerland; ○ Department of Pure and Applied Sciences, University of Urbino, Urbino 61029, Italy; ⧫ Norwegian Institute for Air Research, Kjeller 2007, Norway; †† German Environment Agency (UBA), Wörlitzer Platz, 06844 Dessau-Roßlau, Germany; ‡‡ ICOS Flask and Calibration Laboratory, 28300 Max Planck Institute for Biogeochemistry, Jena 07745, Germany; §§ Hohenpeißenberg Meteorological Observatory, 64386 Deutscher Wetterdienst, Offenbach 63067, Germany; ∥∥ Deutscher Wetterdienst, Offenbach 63067, Germany; ⊥⊥ Department of Meteorology and Geophysics, 27258 University of Vienna, Vienna 1010, Austria

**Keywords:** SF_6_, atmospheric observations, Medusa GC-MS, inverse modeling, emissions, Germany, climate change

## Abstract

Sulfur hexafluoride (SF_6_) is a highly potent greenhouse gas with a Global Warming Potential (GWP) of 24,700 over 100 years and is globally mainly used as an electrical insulator in switchgear. Several measurement networks have tracked SF_6_ for many years and their European data reveal significant emissions in southern Germany. This study focuses on German SF_6_ emissions (2020–2023), using atmospheric measurements from 22 European sites, offering high spatial and temporal resolution for robust emission assessments. While German UNFCCC inventory bottom-up emission estimates report a major source of SF_6_ through the disposal of soundproof windows, the spatial distribution of German SF_6_ emissions derived on top-down inversion techniques (InTEM and Flexinvert+) reveals a different picture: The continuous pattern of high emissions from a particular region is responsible for one-third of total SF_6_ emissions in Germany. Despite this, total German SF_6_ emissions have decreased from 112 ± 26 t in 2020 to 89 ± 15 t in 2023 (InTEM), with estimates from all methods (both bottom-up and top-down) showing similar trends. Our findings suggest that the emissions from soundproof windows are overestimated, while industrial sources - particularly from SF_6_ production and recycling in the focus region - are likely underestimated.

## Graphical abstract

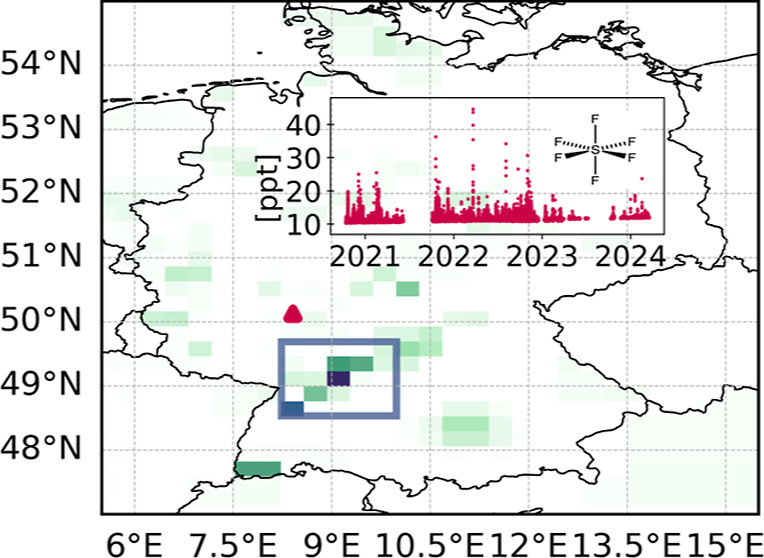

## Introduction

Fluorinated gases have a large impact on global warming because of their high global warming potential (GWP) and long atmospheric lifetimes.^1,2^ Consequently, their production and use are regulated under various international agreements since the 1980s. While some treaties, such as the Montreal Protocol, mandate phase-downs of specific substances, under the United Nations Framework Convention on Climate Change (UNFCCC), countries are required to report emissions and meet overall greenhouse gas reduction targets.^3^ These emission estimates are calculated in accordance with the IPCC (Intergovernmental Panel on Climate Change) methodology guidelines and are based on factors such as production, sales, activity data, emission rates and facility-level measurements.^4–7^ These methods rely on voluntary industry data submissions and assumptions regarding emission factors. As a result, they are accompanied by a high degree of uncertainty, and not all sources are taken into account. Most importantly, illegal or unintentional emissions are not captured by this so-called bottom-up approach. However, emission estimates can also be derived from high-quality, ground-based measurements combined with atmospheric transport models and inversion modeling techniques, which is commonly referred to as the top-down approach. An independent verification of the bottom-up emissions estimates through top-down approaches is not legally binding. The top-down approach is regularly used in some countries, complementing national reporting in order to verify and improve emission estimates.^8,9^ These emission estimates are important not only for monitoring the progress in reducing emissions of greenhouse gases, but also for adjusting policies where necessary. In recent years, numerous studies using inverse techniques have focused on various substances to assess anthropogenic emissions both globally and regionally.^5,10–12^

In this study, we focus on the emissions of sulfur hexafluoride (SF_6_), one of the most potent greenhouse gases currently in use. SF_6_ is an inert gas with strong insulating properties, which accounts for its diverse range of applications. Nowadays, it is globally most commonly used in high-voltage switchgear—a comprehensive summary of applications of SF_6_ has been presented by Simmonds et al. (2020).^13^ Owing to its high Global Warming Potential (GWP_100_) of 24,700 over 100 years^1^ and a long atmospheric lifetime of 850 to 1280 years,^1,14–16^ this fluorinated gas has been increasingly subjected to political regulations and reduction initiatives: SF_6_ was included in the basket of gases under the Kyoto Protocol to reduce parties’ total CO_2_ emissions. As such, many sectors have looked to find more environmentally sustainable alternatives.^17,18^ The European Union’s regulation on fluorinated greenhouse gases also encompasses the reduction of new SF_6_ applications in existing sectors and the encouragement of SF_6_ recycling.^19^

A multitude of studies have investigated the global emission trends of SF_6_ and its primary source regions.^13,20–23^ They agree on increasing global SF_6_ emissions, particularly in Asian countries. With an emission rate of 0.21 Gg yr^–1^(2005–2021), China even surpasses the overall global growth rate of 0.20 Gg yr^–1^.^21^ Several studies have also focused on SF_6_ emissions in Europe, agreeing on the presence of a major source in southern Germany.^13,24^ High emissions from Germany are also reflected in the bottom-up National Inventory Reports (NIR) to the UNFCCC: In 1990, Germany reported emissions of 194.21 t of SF_6_, accounting for 46% of total emissions within the European Union.^25^ To address this, Germany adopted voluntary commitments such as the reuse of SF_6_ in closed-loop systems, monitoring, and research of alternatives^26^ on top of the implementation of the European F-gas regulation.^19^ As a result, Germany reported emissions of 88.61 t of SF_6_ in 2021, corresponding to a reduction of approximately 45% since 1990.^27,28^ The significant reduction in SF_6_ emissions can primarily be attributed to the prohibition of SF_6_ use in vehicle tires, a measure that proved highly effective because of the previous widespread use in Germany.

According to the NIR for 2022, the majority of current SF_6_ emissions originates from the disposal of soundproof windows (68.20%, see Figure 1). These emissions are modeled based on the disposal phase of the windows, assumed to occur after 25 years of use.^29^ In Germany, the use of SF_6_ in soundproof windows was already significantly reduced in the 1990s and it has been banned since 2006.^30^ Windows containing SF_6_ were not labeled, making it impossible to determine their installation locations or track their recycling pathways. Consequently, we expect that the spatial distribution of emissions occur evenly throughout Germany during the disposal of windows, with a focus on large cities. The remaining reported emissions in 2022 are attributed to the following sectors: emissions from switchgear (10.57%), particle accelerators (4.70%), aluminum and magnesium foundries (4.66%) and semiconductor production (3.80%).^27,28^ Other confidentially reported SF_6_ emissions relate to the following areas of application: Radar, welding, optical fibers, shoe soles, medical and cosmetic products, heat transfer agents, solvents and edge insulation in solar cell production (8.05%).^29^ To provide a broader context, Figure 1 illustrates the source composition of SF_6_ emissions in Germany based on the NIR and data from the German Environment Agency between 2010 and 2022. Emissions from the disposal of soundproof windows clearly dominate the bottom-up emission estimates during the review period. However, the German Environment Agency (UBA) assumes a significantly larger reduction in these emissions since 2020. While all other emission sources show a declining trend, the reductions are relatively minor, amounting to only a few tonnes. Despite overall progress, Germany still accounted for 55% of SF_6_ emissions within the European Union in 2022 according to reported emissions to the UNFCCC.^28^ A significant advantage of combining atmospheric observations with inverse modeling techniques is the ability to localize emission sources. Understanding the spatial distribution is crucial for improving emission inventories, validating the bottom-up assumptions, and identifying unknown sources. Observational data from ground-based stations alone provide valuable insights: Long-term data can be used to derive atmospheric background levels, track variability and trends, and reveal regional pollution events. Previous studies investigating SF_6_ emissions in Europe consistently identified a major emission source in southwestern Germany.^13,21,24,31^ In this study, we focus for the first time on the top-down distribution of SF_6_ emissions in Germany, aiming to characterize and quantify the hotspot region in southern Germany. We use observational data from 22 different European atmospheric observation stations in the years 2020 to 2023, in combination with atmospheric transport models and inverse modeling techniques.

**Figure 1:**
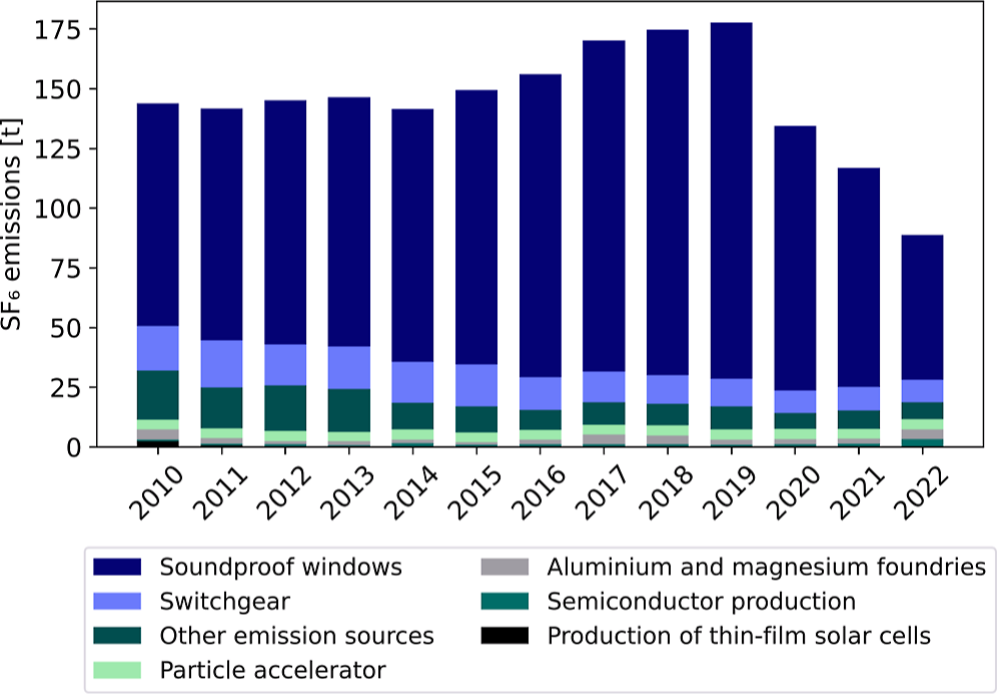
Bottom-up SF_6_ emissions disaggregated by source sector in Germany, based on the NIR^28^ and supplementary data from the German Environment Agency (Umweltbundesamt, UBA),^27^ for the period 2010–2022.

## Methods

### Measurement Data

A sufficiently dense observation network is essential to identify regional sources of halogenated trace gases. This study uses data sets from 22 surface-based observational sites across Europe between 2020 and 2023. A detailed overview is provided in the Supporting Information in Figure 1 and Table 1 and 2. These sites have been measuring SF_6_ for several years and are part of different measurement networks: AGAGE (Advanced Global Atmospheric Gases Experiment), ICOS (Integrated Carbon Observation System), UK DECC Network (Deriving Emissions linked to Climate Change) and GAW (Global Atmosphere Watch).

To expand the AGAGE network in Europe, we started measurements of halogenated trace gases at the Taunus Observatory (TOB) in 2013.^32,33^ TOB is located on top of the mountain Kleiner Feldberg (852 masl) in central Germany. It is a rural site, which is influenced by the surrounding Rhine-Main area. Previous studies showed that this site is sensitive to regional emissions from Germany and potentially also from the Benelux region and France.^32,33^ In this study, we use data from the GC-ECD (Gas Chromatograph-Electron Capture Detector) system between 12 October 2020 and 24 March 2023 based on AGAGE derived calibrations.^34^ The GC-ECD is a component of an airborne measurement system used in several flight campaigns^35–38^ and had a measurement frequency of 20 min when deployed at the observatory. Additionally, we use observational data from the Medusa GC–MS (Gas Chromatograph (Agilent 7890B)-Mass Spectrometer (Agilent 5977B), 2023-02-05 to 2023-12-31), which was installed at TOB in January 2023. The Medusa instrument built by Markes International Ltd. largely follows the setup described in Miller et al. (2008),^39^ which allows for measurements of NF_3_ as an update of the setup described by Arnold et al. (2012).^40^ The instrument uses a Stirling engine to cool the sample traps. Air is sampled at the top of the Kleiner Feldberg mountain from 12 m above ground through a stainless-steel tube (inner diameter: 7.75 mm). Two ambient air measurements are bracketed by measurements of a working standard, thereby providing up to 14 ambient air measurements per day. The measurements of the TOB Medusa are calibrated and evaluated using the AGAGE procedures.^41^ The SF_6_ data are reported on the SIO-05 calibration scale.

In addition to TOB, there are five other active AGAGE sites across Europe, all equipped with a Medusa preconcentration unit and a GC–MS:^42–44^ Mace Head (MHD, Ireland), Tacolneston (TAC, United Kingdom), Jungfraujoch (JFJ, Switzerland), Monte Cimone (CMN, Italy), and Zeppelin (ZEP, Svalbard).^44^ These measurements are all reported on the SIO-05 calibration scale.^41^

For this publication, we used three of the five UK DECC network sites: The SF_6_ measurements from Ridge Hill (RGL), Bilsdale (BSD) and Heathfield (HFD) are made using GC-ECD, have a temporal resolution of 20 min and are based on the AGAGE-derived SIO-05 calibration scale.^45–47^ TAC and MHD are both part of the AGAGE and UK DECC networks. In this study, data from the AGAGE network was used from the two stations. In addition, we used a site from the GAW Program located in the south of Germany. The Environmental Research Station Schneefernerhaus (ZSF) is situated 312 m below the summit of the highest German mountain, Zugspitze (2962 masl). Using a GC-ECD, SF_6_ has been measured at ZSF for more than 15 years and is calibrated against the NOAA calibration scale WMO SF_6_ X2014.^21^

The ICOS network is one of the largest pan-European research infrastructures. ICOS RI (Integrated Carbon Observation System Research Infrastructure) provides high-quality measurement data of greenhouse gases and support the member countries in their national inventory reports and climate mitigation strategies.^48^ SF_6_ flask samples are collected at class 1 atmospheric stations. In this study, we used the data from 11 sites that were downloaded from the ICOS carbon portal: Cabauw (CBW, Netherlands^49^), Gartow (GAT, Germany^50^), Hohenpeissenberg (HPB, Germany^51^), Hyltemossa (HTM, Sweden^52^), Karlsruhe (KIT, Germany^53^), Lindenberg (LIN, Germany^54^), Norunda (NOR, Sweden^55^), Observatoire pérenne de l’environment (OPE, France^56^), Ochsenkopf (OXK, Germany^57^), Pallas (PAL, Finland^58^), Saclay (SAC, France^59^), Steinkimmen (STE, Germany^60^). The samples collected at these sites are analyzed at the ICOS Flask and Calibration Laboratory in Jena (Germany) and calibrated against the NOAA scale (WMO SF_6_ X2014).

In CBW, a second set of flask samples was taken on the ICOS sampler by The Netherlands Organisation for Applied Scientific Research (TNO) and analyzed by the University of Bristol on a laboratory based Medusa GC–MS system at AGAGE derived SIO-05 scale.^34^ Together with the flasks sampled for ICOS this results in a sampling frequency of one flask a day for CBW.

All data originally reported on the WMO SF_6_ X2014 calibration scale were converted to the SIO-05 scale using published conversion factors (1.0049 ± 0.0029 ^41^). High SF_6_ mole fractions that are observed in pollution events occasionally exceed by far the calibrated range set by the NOAA WMO SF_6_ X2014 scale (2–20 ppt). The uncertainties are derived from the repeatability and variability of the standard measurements. Higher mole fractions of a measurement correspond to a larger absolute error, ensuring that the reduced reliability of data outside the calibration range is appropriately reflected. More detailed information on location, measurement frequency and measurement precision at the sites can be found in Table 1 and 2 in the Supporting Information.

### Inverse Modeling Techniques and Emission Inventories

To quantify German SF_6_ emissions, we combined the Lagrangian atmospheric transport model NAME (Numerical Atmospheric dispersion Modeling Environment^61–63^ ) with the Bayesian optimization framework InTEM (Inversion Technique for Emission Modeling^62,64^ ). These two models have already been used in numerous studies focusing on different trace gases and regions.^10,62,65,66^ NAME releases 20,000 particles at each site every hour. Using the three-dimensional meteorological Unified Model with a resolution of 12 km, these particles are tracked 30 days backward in time, or until they leave the computational domain,^5,63^ to create source receptor relationships. In NAME, SF_6_ is treated as a passive tracer without loss processes. This assumption is consistent with the long atmospheric lifetime of SF_6_.^1,14,15^ In the next step, InTEM uses the NAME output from each site to minimize the difference between the computed mole fractions and actual atmospheric observations at the 22 sites. This process is repeated 24 times each time with 10% of the observations removed.^5^ Regions in InTEM are initially defined in alignment with national borders and they are further divided within the modeling, based on the impact the different grid cells have at the observation sites, see Manning et al. (2021)^5^ for a detailed explanation. An important part of the modeling process is the determination of baseline mole fractions, as it is the perturbation above baseline at each site that provides the signal with which to infer regional emissions. For this study, initial baseline mole fractions were calculated (as described in Manning et al. (2021)^5^) at MHD, JFJ, CMN and ZEP, with the remaining sites using the MHD baseline. Adjustments from the 11 boundary regions surrounding the computational domain then result in unique baselines at each site, with each station having a further freedom within the modeling to allow a small bias from other sites; the methodology is described in greater detail in Arnold et al. (2018).^63^ The EDGAR v8 (Emissions Database for Global Atmospheric Research—EDGARv8.0) country totals were evenly distributed across each country as the prior emission value. EDGAR uses a technology-based emission factor approach that utilizes country-specific activity data, the mix of technology in each sector, abatement measures, emission factors and the reduction by abatement to estimate the emissions of various greenhouse gases and ozone-depleting substances.^67–70^ Emission estimates from InTEM were derived for three-month periods: January to March, April to June, July to September, and October to December and the results were averaged to determine the annual SF_6_ emissions for the years 2020 to 2023. To investigate the high-emission region in greater detail, we defined a focus region (48.637 °N to 49.807 °N, 8.404 °E to 10.164 °E). Furthermore, we conducted two additional runs with InTEM to analyze the effect of the eight German stations. Specifically, one run without the two most polluted sites (TOB and KIT) and another excluding all German monitoring sites. The results of the InTEM inversions with all 22 European sites are compared with German SF_6_ emissions reported to the UNFCCC and with the data from EDGAR (EDGAR_2024_GHG^70^).

Vojta et al. (2025) recently published a study focusing on European SF_6_ emissions for the period 2005–2021.^31^ They investigated European SF_6_ emissions using the Lagrangian particle dispersion model FLEXPART (FLEXible PARTicle dispersion model)^71^ and the Bayesian inversion framework Flexinvert+.^72^ This provides an opportunity to compare our InTEM results with those from a completely independent atmospheric transport and inversion model, also using different a priori emissions, meteorological input and SF_6_ measurement data sets. Here, we use the setup as used in Vojta et al. (2025) but do not only compare the total German emissions for 2020 and 2021, but also examine the results for a similar focus region over the high-emission region in southwestern Germany (48.75 °N to 50.0 °N, 8.25 °E to 10.00 °E) as defined for InTEM.^31^ Note that for the FLEXPART/Flexinvert+ inversions, the same global data set as in Vojta et al. (2024) was used. Therefore, only three German sites (OXK, HPB and ZSF) were included in the inversion.^21^

## Results and Discussion

### Frequent Pollution Events of SF_6_ in Southwest Germany

In this study, we extend previous research by incorporating newly available SF_6_ data sets from Germany and other parts of Europe, along with, for the first time, high-resolution data from the Taunus Observatory (TOB). Compared to other German monitoring sites, the TOB data set, alongside the KIT data set, stands out: both stations regularly detect high SF_6_ mole fractions, indicating regional emissions (see Supporting Information Figure 2). Other sites in northern (STE, GAT, LIN), eastern (OXK), and southern (HPB, ZSF) Germany rarely observe such events. An averaged NAME footprint for the year 2023 for each German site is provided in Figure 4 in the Supporting Information to provide a better understanding of the typical source regions influencing each station.

Figure 2 A shows a representative excerpt from the measurement time series at TOB between 2022-10-15 and 2022-11-15 with frequent pollution events. The data are shown as 4-hly averages to allow comparison with InTEM, which operates at a 4 h resolution. To differentiate between background levels and pollution events, a baseline filter is applied to observed mole fractions at TOB. The baseline describing the background levels results from a fit function that best describes the measurement data. The fit parameters are adjusted until the 2-fold standard deviation (2 σ) changes by less than 10% by removing data points above the 2 σ range step by step. After the final step, data points within the 2 σ range around the fitted baseline are categorized as background levels. Data points outside of this 2 σ range are defined as polluted.^32^ Across the entire observation data set at TOB, 82% of the data correspond to background levels and 18% to pollution events.

**Figure 2:**
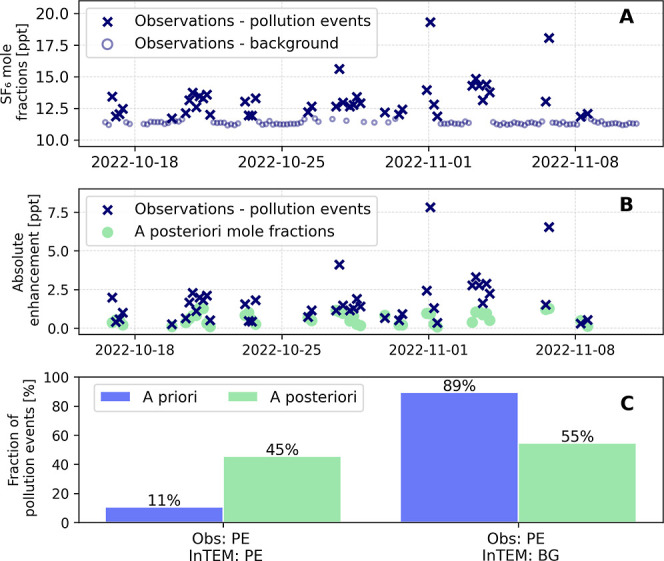
A: Representative excerpt from the SF_6_ measurement time series at TOB between 2022-10-15 and 2022-11-15. Blue circles represent background mole fractions, and dark blue x symbols indicate pollution events. The classification into background and pollution event is based on the statistical filter described in Schuck et al. (2018).^32^ The data are shown as 4-hly averages to allow direct comparison with InTEM, which operates at a 4 h resolution. B: Comparison of absolute enhancements above the baseline identified with the baseline filter^32^ for statistical identified pollution events from the time series in Panel A. Observations (dark blue x) and posterior mole fractions (light green) from InTEM are displayed. C: Comparison between the full observational data set (Obs.) over the entire study period and the modeled prior (light blue)/posterior (light green) InTEM data set. The left bars show the fraction of pollution events (PE) in the observations that were also identified as pollution events in the modeled prior/posterior in InTEM. The right bars show the fraction of pollution events in the observations that were identified as background (BG) in InTEM.

InTEM initially distributes the assumed prior emission from EDGAR evenly across each country and region. The posterior mole fractions are derived by assimilating observational data into the model, thereby adjusting the prior estimates. Figure 2 B shows the enhancements of the pollution events (from Panel A) in the observational data, calculated as the deviations above the fitted baseline using the described statistical filter.^32^ For comparison, the corresponding posterior data is plotted for each observation. It becomes evident that the modeled posterior data at TOB cannot reproduce the magnitude of the high pollution events. In contrast, lower-intensity events are represented reasonably well by InTEM. To assess how the full data set behaves, we compared in Figure 2C how the modeled prior and posterior data sets represent the pollution events identified in the observations (18% of the whole data set). In the prior, only 11% of the observed pollution events are reproduced, whereas in the posterior this fraction increases to 45%. Noting the fraction of pollution events in the observational data set that are incorrectly considered background in InTEM, we also see a clear improvement: from 89% in the prior to 55% in the posterior data set. When comparing the complete data sets, the correlation coefficient improves from 0.48 (prior mole fractions) to 0.63 (posterior mole fractions) (Supporting Information Figure 5) and the root-mean-square error improves from 0.62 ppt (prior) to 0.57 ppt (posterior).This analysis shows that there is a clear improvement from the prior to the posterior, however the posterior data set is not always able to capture the full magnitude of the measured pollution events. Consequently some lower-intensity pollution events in the posterior are not always identified by the statistical filter as the absolute enhancement above the fitted baseline is too small. Several factors contribute to this behavior: first, we use three-month averaged emission estimates, which smooths intermittent high pollution events. Second, emissions within an inversion grid are assumed to be uniformly distributed and constant, which can also prevent high pollution events from being represented sharply and at the observed magnitude. Of course, the resolution of the meteorological model (Unified Model, ∼12 km), the atmospheric transport model (NAME, ∼ 25 km) and the inversion model (InTEM, ∼25 km) plays a significant role in determining how accurately mole fractions at a given site can be reproduced. A more detailed analysis of NAME and InTEM can be found in Arnold et al. (2018).^63^ Although the observed high pollution events are not captured at the full magnitude in the InTEM results, they are still registered and InTEM provides a realistic representation of the regional emission patterns. However, since InTEM does not overestimate the high pollution events, this suggests that the posterior emissions may still be underestimated, and that the real emissions could be higher.

### SF_6_ Emissions in Germany: Spatial Distribution and Comparison with Bottom-Up Estimates

To gain insights into the origin of air masses associated with the pollution events, hourly averaged NAME air history footprints are generated. For TOB, the air history at the highest data point of the excerpt in Figure 2 shows a pollution event originating from the south and southeast of Germany (see Supporting Infomation Figure 3). With the high density of observations in Germany and Europe used in this study, it was possible to achieve a higher spatial resolution of SF_6_ emissions in Germany, compared to previous studies. This is supported by two additional runs with InTEM: one without the measurements of the two most polluted sites (TOB and KIT) and another excluding all German monitoring sites. These experiments demonstrate that reducing the number of observational sites diminishes, but does not remove, the model’s ability to identify the point source. It simply decreases the accuracy of the location in the southwest of Germany and increases the variability of the total emission estimates. The results of these additional runs are presented in the Supporting Information (Table 3, Figure 6, Figure 7).

Figure 3 shows the annually averaged SF_6_ emission map for Germany, covering the period from 2020 to 2023, derived from measurements at all 22 observational sites. The high-emission region in southwestern Germany is a persistent feature observed every year. The highest posterior emission value of a grid cell estimated ranges between 23.4 kg yr^–1^ km^–2^ (2020) and 13.5 kg yr^–1^ km^–2^ (2021). The reliability of our findings is highlighted by the consistency of the emission patterns produced by FLEXPART and Flexinvert+, despite the use of different models and data sets (Supplementary Information Figure S8). In particular, most of the German stations, including TOB, were not used in this inversion. In addition to these to top-down approaches, we also examined the gridded EDGAR SF_6_ emissions, which indicate higher emission estimates around larger cities. An averaged emission map of Germany for the period 2020–2023, based on the EDGAR estimates, is provided in the Supporting Information (see Figure 9).

**Figure 3:**
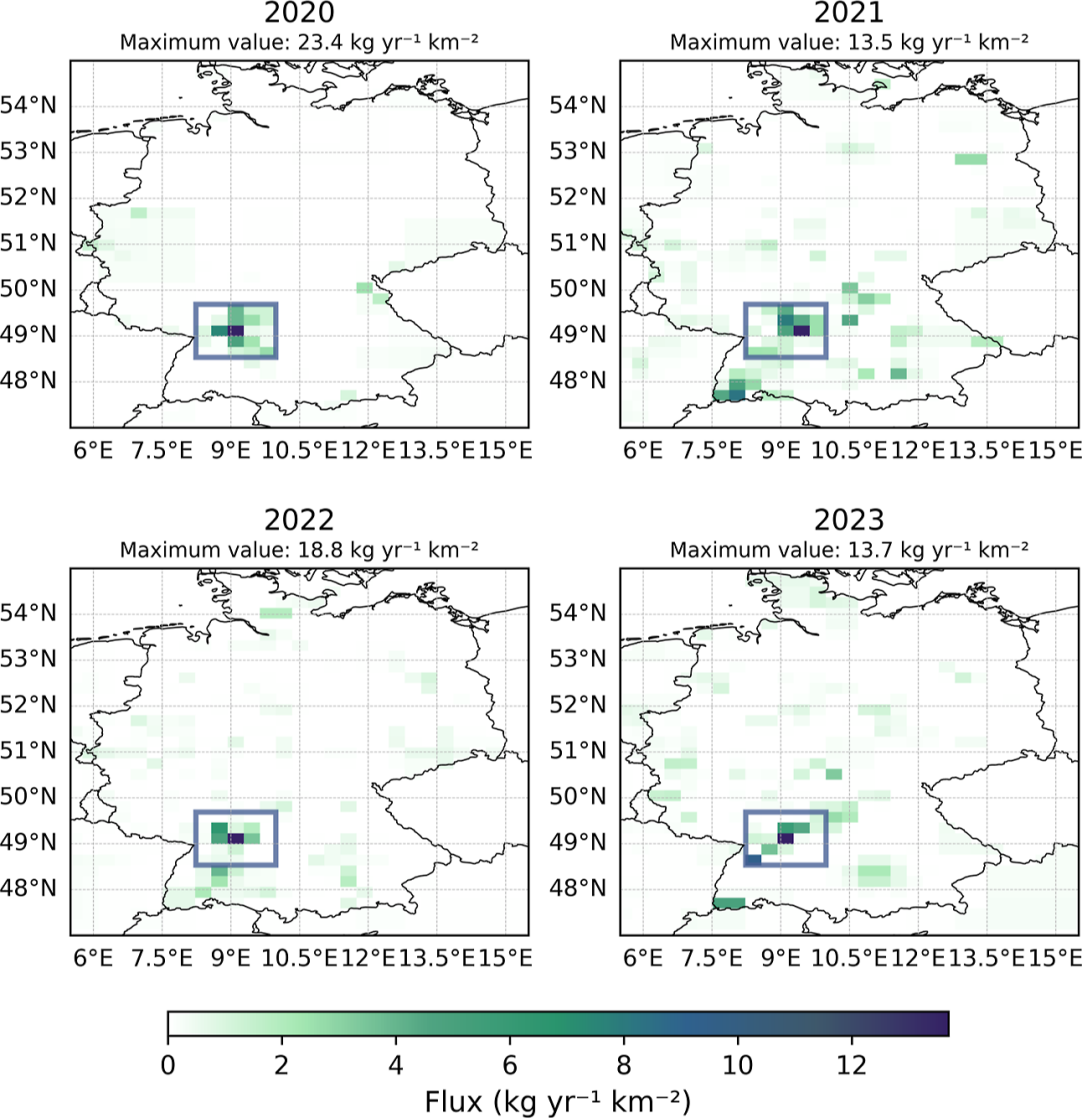
Annual averaged InTEM top-down inversion emission estimates for SF_6_ (Flux kg yr^–1^ km^–2^) for the period 2020–2023 with the focus on Germany. A focus region, shown in the figures as a blue box (48.637 °N to 49.807 °N, 8.404 °E to 10.164 °E), was defined and analyzed to characterize emissions in the high emission region. The highest emission value of a grid cell in each respective year is shown in the figure.

The uncertainty of the emission estimates in InTEM from the prior to the posterior has been significantly improved: We observe a substantial reduction in flux uncertainty, from 200% defined for the prior emission field to approximately 18% for the posterior emission field. Figure 4 compares total SF_6_ emissions reported by Germany to the UNFCCC (2020–2022), estimates by EDGAR (EDGAR_2024_GHG, 2020–2023 ^70^), the top-down inversion results from FLEXPART/Flexinvert+ (2020–2021) and NAME/InTEM (2020–2023). In 2020, all four methods estimated Germany’s total SF_6_ emissions to exceed 100 t (EDGAR: 139 t, UNFCCC: 132 t, Flexinvert+: 119 ± 20 t, InTEM: 112 ± 26 t), but by 2023, emissions had declined to 76 t (EDGAR) and 89 ± 15 t (InTEM), demonstrating a consistent downward trend across all emission estimates.

**Figure 4:**
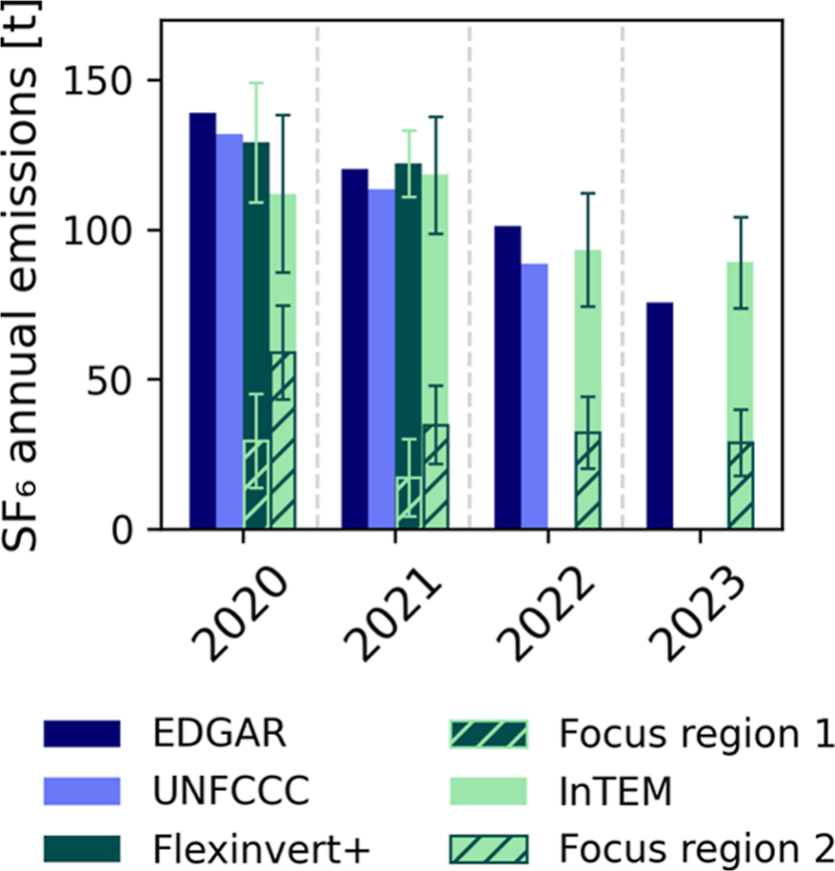
German SF_6_ emission estimates [*t* yr^–1^] from EDGAR (2020–2023),^70^ UNFCCC inventory (2020–2022),^28^ Flexinvert+ (2020–2021)^31^ with focus region 1 (48.75 °N to 50.0 °N, 8.25 °E to 10.00 °E) and InTEM (2020–2023) with focus region 2 (48.637 °N to 49.807 °N, 8.404 °E to 10.164 °E). The error bars describe the 2 σ uncertainty of the ensemble distribution for Flexinvert+ and the InTEM inversion results.

Comparing the two top-down methods, Flexinvert+ estimates higher total German SF_6_ emissions than InTEM, with smaller differences in 2020 (∼7 t) compared to 2021 (∼23 t), which are in the range of the uncertainties of the models. An additional region was defined for both inversion runs in order to quantify the emissions of the high emission area. Due to the different model resolutions, the defined focus regions are not identical (focus region 1 in Flexinvert+: [48.75 °N to 50.0 °N, 8.25 °E to 10.00 °E]—Supporting Information Figure 8, focus region 2 in InTEM: [48.637 °N to 49.807 °N, 8.404 °E to 10.164 °E]—Figure 3). Emissions within these regions are represented by the hatched areas of the respective bars in Figure 4. In focus region 1, Flexinvert+ estimates emissions that are approximately half as high as those estimated by InTEM in focus region 2 for 2020 and 2021: In 2020, Flexinvert+ calculated emissions of 28 ± 9 t, whereas InTEM estimated 59 ± 16 t, and in 2021, Flexinvert+ estimated an even lower emission of 17 ± 5 t. A decline in this focus region is also observed in the InTEM results, with emissions decreasing from 35 ± 13 t in 2021 to 32 ± 12 t in 2022 and 29 ± 11 t in 2023. The discrepancies between the two top-down methods can partly be explained by differences in the monitoring sites used for each inversion: The two sites TOB and KIT play a key role in refining emission estimates and are only included in the InTEM inversions. These sites are located in regions with frequent and high-level pollution events, providing valuable observational data that enhance InTEM’s sensitivity to emissions in the focus region and improve the spatial attribution across Germany and neighboring countries. Further evidence is provided by the two additional InTEM runs, one performed without TOB and KIT and another excluding all German sites, presented in the Supporting Information (Table 3, Figure 6, Figure 7).

Top-down inversion techniques offer the advantage of combining information on total and regional emission magnitudes with their spatial distribution. The emissions reported from the bottom-up approach show that the majority of SF_6_ emissions in Germany are attributable to emissions from the disposal of soundproof windows (Figure 1). Between 2020 and 2022, this ranges between 68% (2022) and 82% (2020) of the total German emissions. As this is the dominant source and soundproof windows are mainly used in and around large cities, an emission distribution with slightly higher emissions in the vicinity of large cities would be assumed. The remaining share of German bottom-up emissions is distributed among many other smaller sources. On this basis, the spatial distribution of top-down emission estimates (InTEM Figure 3, Flexinvert+ Supporting Information Figure 8) and particularly the high emissions in southwest Germany cannot be explained.

The agreement between total emissions from bottom-up reports and top-down emission estimates from our study suggests that either certain sources were not taken into account or underestimated, while emissions of other sources were overestimated in the bottom-up inventory: According to the InTEM calculations, 37% of total German emissions were emitted on average in the focus region between 2020 and 2023. Furthermore, Flexinvert+ emission estimates in this area are broadly consistent with those of InTEM. Consequently, it can be deduced that the calculated emission estimates from soundproof windows have been significantly overestimated. This suggests either that these windows remain in use for a longer period, leading to a larger stock of SF_6_ in Germany and prolonging its emissions and their environmental impact. Or the windows have already been replaced and SF_6_ has already been emitted.

Further investigations revealed that the only factory currently producing and recycling SF_6_ in Europe is located within the area where high emissions were estimated. Although estimating emissions in our relatively large focus area may include contributions from multiple sources, these high emissions cannot be explained by diffusive sources like window disposals. This is an indication that fugitive emissions during the manufacturing and recycling of SF_6_ have been significantly underestimated.

We conclude that bottom-up SF_6_ emission inventories do not accurately capture regional emission patterns in Germany. Our study has demonstrated that top-down estimates are essential to correct these patterns and even identify sources missing from these inventories. The necessity of MRV systems (Monitoring, Reporting, Verification) and their success has been clearly demonstrated by numerous studies. While MRV systems cannot and should not replace bottom-up inventory reports, they are essential for improving and validating these reports and the associated policies. They not only validate reported emissions but also help identify unknown, unreported, or even illegal emissions. Two examples where observations and inversions, in conjunction with political or corporate engagement, have led to the mitigation of emissions of ozone-depleting substances and potent greenhouse gases are highlighted in the studies on CFC-11 by Montzka et al. (2018)^73^/Rigby et al. (2019)^65^ and on methane emissions by Kuhlmann et al. (2024).^74^ Another advantage of top-down emission estimates is their near real-time availability: most bottom-up reports are delayed by up to two years due to the complex reporting system. Therefore, observational-based top-down methods, such as NAME/InTEM or FLEXPART/Flexinvert+, can provide faster feedback on new policy strategies. Furthermore, seasonal or production-related emission patterns can only be detected through observational-based inversions.

In conclusion, these findings underscore the importance of refining emission inventories through both bottom-up and top-down approaches to provide more accurate emission data. The integration of MRV systems will play a key role in ensuring timely policy adjustments and fostering more effective emission mitigation strategies. As we move forward, further research into the lifecycle of soundproof windows and more comprehensive assessments of regional emission sources will be crucial in improving Germany’s SF_6_ emission assessments and ultimately reducing emissions. This also requires continuing the dialogue with industry, political decision-makers and the administration. The underlying goal should be to work together to ensure that unintended emissions are reduced during the production and recycling of SF_6_.

## Supplementary Material



## Data Availability

The SF_6_ measurement data underlying this study are openly available from the CEDA Archive at https://catalogue.ceda.ac.uk/uuid/040f19261fa24683988bff79b255f0a8/?jump=related-docs-anchor.^47^ (UK DECC Network), from the AGAGE data archive at https://www-air.larc.nasa.gov/missions/agage/data/version-history/20250123.^75^ (AGAGE), from the ICOS Data Portal at https://data.icos-cp.eu/portal/, from the World Data Centre for Greenhouse Gases at https://gaw.kishou.go.jp/and the SF_6_ data at the sites TOB (ECD) and CBW is available on the ICOS carbon portal at https://meta.icos-cp.eu/objects/oAzNtfjXddcnG_irI8fJT7W6.^34^

## Notes

Station locations and metadata (network information, calibration scales, sampling height, measurement precision and frequency), NAME footprints of one representative pollution event and averaged NAME air history maps for all German sites for 2023, correlation plots between InTEM data and atmospheric observations, more details about the additional InTEM runs, information on the FLEXPART/Flexinvert+ inversions and the averaged gridded EDGAR emission distribution of SF_6_ over Germany (PDF)

## Abbreviations

AGAGE: Advanced Global Atmospheric Gases Experiment
BSD: Bilsdale (United Kingdom)
CBW: Cabauw (Netherlands)
CMN: Monte Cimone (Italy)
EDGAR: Emissions Database for Global Atmospheric Research
FLEXPART: Flexible Particle dispersion model
GAT: Gartow (Germany)
GAW: Global Atmosphere Watch
GC–ECD: gas chromathograph–electron capture detector
GC–MS: gas chromathograph–mass spectrometer
GWP: global warming potential
HFD: Heathfield (United Kingdom)
HPB: Hohenpeissenberg (Germany)
HTM: Hyltemossa (Sweden)
ICOS (RI): Integrated Carbon Observation System (Research Infrastructure)
InTEM: Inversion Technique for Emission Modeling
IPCC: Intergovernmental Panel on Climate Change
JFJ: Jungfraujoch (Switzerland)
KIT: Karlsruhe (Germany)
LIN: Lindenberg (Germany)
MHD: Mace Head (Ireland)
MRV: monitoring, reporting, validating
NAME: numerical atmospheric dispersion modeling environment
NIR: National Inventory Reports
NOAA: National Oceanic and Atmospheric Administration
NOR: Norunda (Sweden)
OPE: Observatoire ṕerenne de l’environment (France)
OXK: Ochsenkopf (Germany)
PAL: Pallas (Finland) ppt parts per trillion
RGL: Ridge Hill (United Kingdom)
SAC: Saclay (France)
STE: Steinkimmen (Germany)
TAC: Tacolneston (United Kingdom)
TNO: Nederlandse Organisatie voor Toegepast Natuurwetenschappelijk Onderzoek
TOB: Taunus Observatory (Germany)
UBA: Umweltbundesamt - German Environment Agency
UK DECC: United Kingdom - Deriving Emissions linked to Climate Change
UNFCCC: United Nations Framework Convention in Climate Change
WMO: World Meteorological Organisation
ZEP: Zeppelin (Norway)
ZSF: Zugspitze (Germany)
