# Supplementary material for: Characterization of German SF6 Emissions
Source: ACS EST Air. 2025 Nov 6;2(12):2889–99. doi: 10.1021/acsestair.5c00234 (PMC12706706; doi:10.1021/acsestair.5c00234)
Supplement: Supplementary file 1 [file ea5c00234_si_001.pdf]

Supporting Information for: Characterisation of German SF_6 emissions

Katharina Meixner,[*,†] Thomas Wagenhäuser,[†] Tanja J. Schuck,[†] Sascha Alber,[‡] Alistair J. Manning,[¶] Alison L. Redington,[¶] Kieran M. Stanley,[§] Simon O'Doherty,[§] Dickon Young,[§] Joseph Pitt,[§] Angelina Wenger,[§] Arnoud Frumau,[‖] Ann R. Stavert,[⊥] Christopher Rennick,[#] Martin K. Vollmer,[@] Michela Maione,[△] Jgor Arduini,[△] Chris R. Lunder,[∇] Cedric Couret,[††] Armin Jordan,[‡‡] Xochilt Gutiérrez Gutiérrez,[‡‡] Dagmar Kubistin,[¶¶] Jennifer Müller-Williams,[§§] Matthias Lindauer,[§§] Martin Vojta,[⊥⊥] Andreas Stohl,[‖‖] and Andreas Engel[†]

E-mail: meixner@iau.uni-frankfurt.de

Station locations and meta data

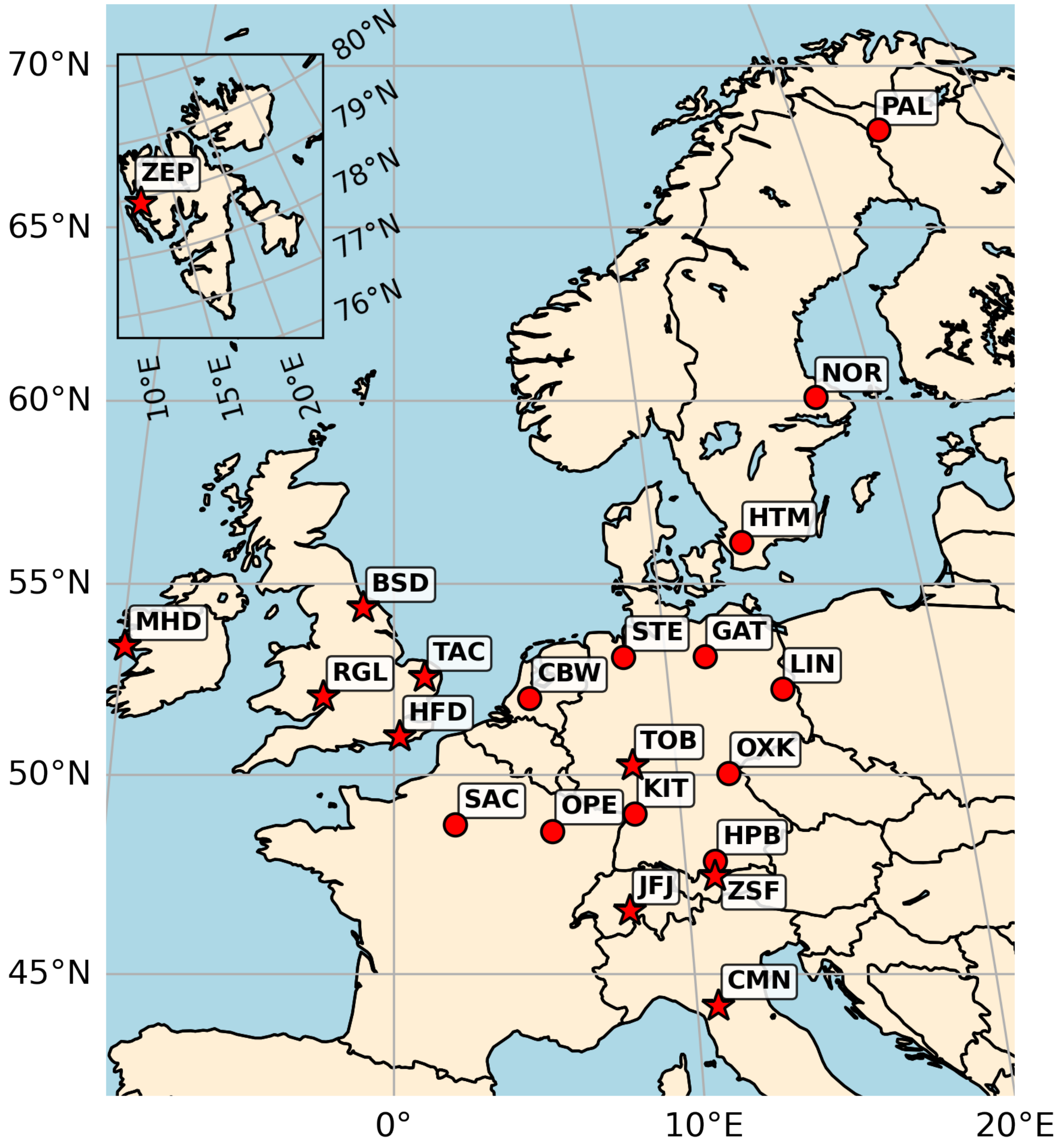

Figure 1: Locations of the monitoring stations used in this study. Stations with continuous measurements are indicated with red stars (BSD, CMN, HFD, JFJ, MHD, RGL, TAC, TOB, ZEP, ZSF), stations with flask measurements are indicated with red circles (CBW, GAT, HPB, HTM, KIT, LIN, NOR, OPE, OXK, PAL, SAC, STE). Coordinates of all sites are listed in Table 1.

Table 1: Overview of the measurement stations used in this study, their abbreviations, coordinates and geographical height.

Site		Country	Latitude	Longitude	Geographical height [m a.s.l.]
BSD	Bilsdale	United Kingdom	54.3368 °N	1.1334 °W	380
CBW	Cabauw	Netherlands	51.9703 °N	4.9264 °E	0
CMN	Monte Cimone	Italy	44.1936 °N	10.6999 °E	2165
GAT	Gartow	Germany	53.0657 °N	11.4429 °E	70
HFD	Heathfield	United Kingdom	50.9698 °N	0.2310 °E	160
HPB	Hohenpeissenberg	Germany	47.8011 °N	11.0246 °E	934
HTM	Hyltemossa	Sweden	56.0976 °N	13.4189 °E	115
JFJ	Jungfraujoch	Switzerland	46.5475 °N	7.9851 °E	3559
KIT	Karlsruhe	Germany	49.0915 °N	8.4249 °E	110
LIN	Lindenberg	Germany	52.1663 °N	14.1226 °E	73
MHD	Mace Head	Ireland	53.3267 °N	9.9046 °W	8
NOR	Norunda	Sweden	60.0864 °N	17.4794 °E	46
OPE	Observatoire pérenne de l'environment	France	48.5619 °N	5.5036 °E	390
OXK	Ochsenkopf	Germany	50.0300 °N	11.8083 °E	1022
PAL	Pallas	Finland	67.9733 °N	24.1157 °E	565
RGL	Ridge Hill	United Kingdom	51.9975 °N	2.5399 °W	204
SAC	Saclay	France	48.7227 °N	2.1420 °E	160
STE	Steinkimmen	Germany	53.0431 °N	8.4588 °E	29
TAC	Tacolneston	United Kingdom	52.5188 °N	1.1387 °E	56
TOB	Taunus Observatory	Germany	50.2219 °N	8.4471 °E	825
ZEP	Zeppelin	Norway	78.907 °N	11.889 °E	474
ZSF	Zugspitze	Germany	47.4165 °N	10.9796 °E	2666

Table 2: Overview of the measurement stations used in this study: associated networks, calibration scales, inlet and InTEM particle release heights, complete measurement period, precision and frequency.

Site	Network	Scale	Sampling height [magl]	Release height [magl]	Measuring period	measurement precision	measurement frequency
BSD	UK DECC	SIO-05 [a]	248	248	2014-01-30 – 2021-08-10	0.047 ppt	5 h^{-1}
CBW	ICOS	WMO SF_6 X2014	207	200	2021-11-04 – 2023-12-17	0.035 ppt	1 d^{-1}
	–	SIO-05 [a]	207	200	2021-02-14 – 2024-02-23	0.6 % [e]	
CMN	AGAGE	SIO-05	7	10 [d]	2023-09-16 – 2023-10-16	0.6 % [e]	11 d^{-1}
GAT	ICOS	WMO SF_6 X2014	341	341	2021-07-01 – 2023-11-12	0.035 ppt	3 w^{-1}[f]
HFD	UK DECC	SIO-05 [a]	100	100	2014-01-01 – 2023-12-31	0.040 ppt	5 h^{-1}
HPB	ICOS	WMO SF_6 X2014	131	131	2019-07-18 – 2023-12-28	0.035 ppt	3 w^{-1}[f]
HTM	ICOS	WMO SF_6 X2014	150	150	2020-03-24 – 2023-12-14	0.035 ppt	3 w^{-1}[f]
JFJ	AGAGE	SIO-05	2	10 [c]	2007-12-07 – 2024-08-11	0.6 % [e]	11 d^{-1}
KIT	ICOS	WMO SF_6 X2014	200	200	2019-06-26 – 2023-11-21	0.035 ppt	3 w^{-1}[f]
LIN	ICOS	WMO SF_6 X2014	98	98	2020-08-11 – 2023-12-16	0.035 ppt	3 w^{-1}[f]
MHD	AGAGE	SIO-05	10	10	2003-05-22 – 2024-08-12	0.6 % [e]	11 d^{-1}
NOR	ICOS	WMO SF_6 X2014	100	100	2019-07-09 – 2023-11-01	0.035 ppt	3 w^{-1}[f]
OPE	ICOS	WMO SF_6 X2014	120	120	2016-02-25 – 2023-12-05	0.035 ppt	3 w^{-1}[f]
OXK	ICOS	WMO SF_6 X2014	163	163	2021-04-22 – 2024-01-02	0.035 ppt	3 w^{-1}[f]
PAL	ICOS	WMO SF_6 X2014	12	10	2020-10-31 – 2023-12-24	0.035 ppt	3 w^{-1}[f]
RGL	UK DECC	SIO-05 [a]	90	90	2012-03-01 – 2023-12-31	0.044 ppt	5 h^{-1}
SAC	ICOS	WMO SF_6 X2014	100	100	2019-05-15 – 2023-10-15	0.035 ppt	3 w^{-1}[f]
STE	ICOS	WMO SF_6 X2014	252	252	2021-06-30 – 2023-12-19	0.035 ppt	3 w^{-1}[f]
TAC	AGAGE	SIO-05	185	185	2012-01-17 – 2024-08-12	0.033 ppt	11 d^{-1}
TOB	–	SIO-05 [b]	12	10	2020-10-12 – 2023-03-24	0.100 ppt	3 h^{-1}
	AGAGE	SIO-05	12	10	2023-02-05 – 2023-12-31	0.050 ppt	14 d^{-1}
ZEP	AGAGE	SIO-05	15	10	2010-09-03 – 2024-08-12	0.6 % [e]	11 d^{-1}
ZSF	GAW	WMO SF_6 X2014	3	600	2014-04-01 – 2023-12-31	0.09 ppt	1 h^{-1}[g]

[a] AGAGE derived calibration scale: For these measurements, a standard flask calibrated on the AGAGE scale at an AGAGE station was provided. [b] ECD measurements were operated on AGAGE derived scale. [c] Actual release height at 1000 m due to high altitude. [d] Actual release height at 500 m due to high altitude. [e] The general measurement precision of the Medusa setup for SF_6 was defined by Prinn et al. (2018).[1] [f] ICOS flasks were collected at varying intervals, resulting in an anverage fo three samples per week. [g] The ECD setup has a measurement frequency of 5-8 measurements per hour. The hourly mean values were used in this study.

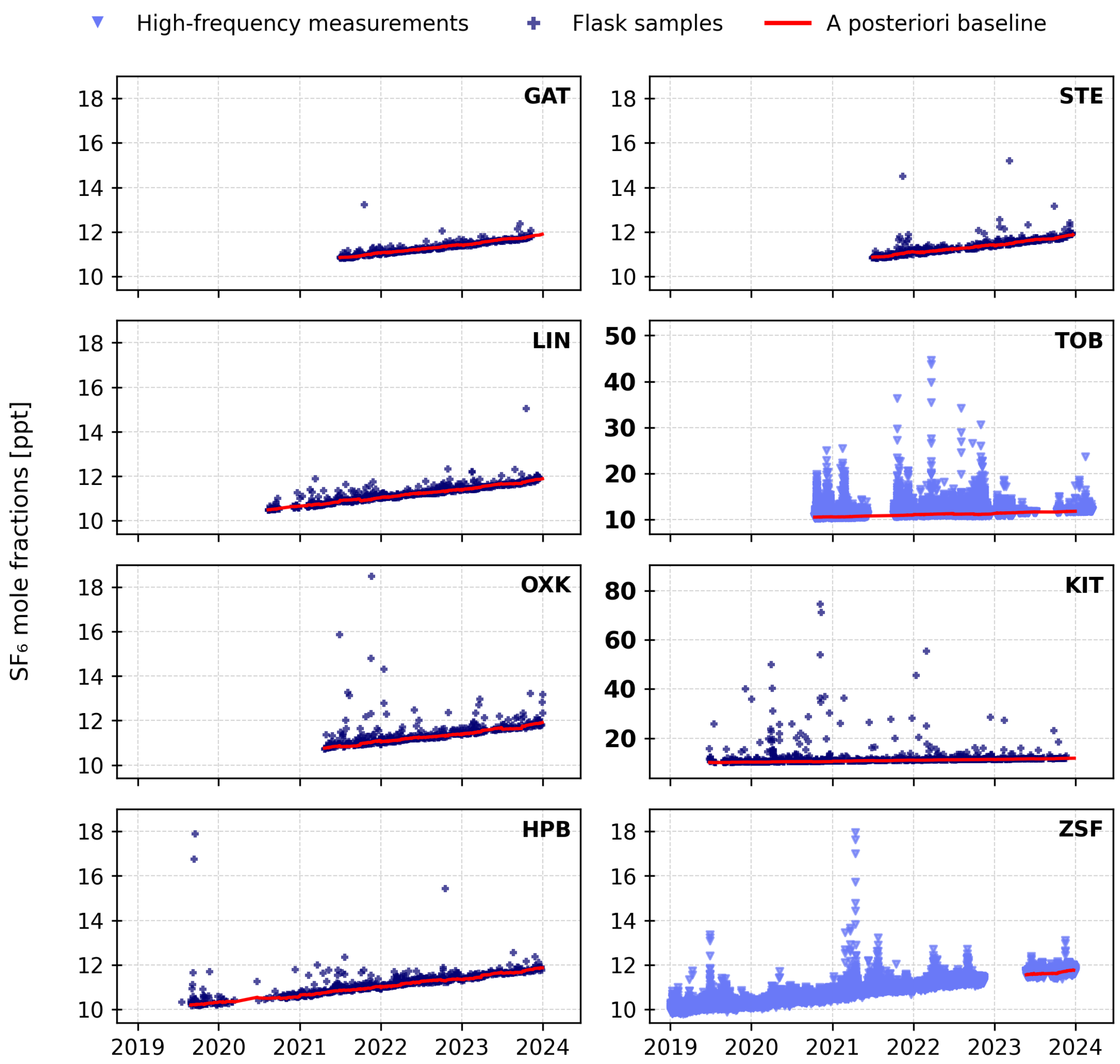

Figure 2: Time series of SF_6 mole fractions (ppt) at all German monitoring stations (01/2019 – 03/2024). Light blue inverted triangles indicate continuous measurements (TOB - Taunus Observatory, ZSF - Zugspitze); dark blue pluses represent flask samples (STE - Steinkimmen, GAT - Gartow, LIN - Lindenberg, OXK - Ochsenkopf, KIT Karlsruhe, HPB - Hohenpeissenberg). For all sites except TOB and KIT, a common y-axis range was used. Due to higher pollution events at TBO and KIT, theses sites use a different y-axis range, with their y-axis labels emphasised in bold. The posterior baseline (InTEM) at each site is shown as a red line.

NAME footprints

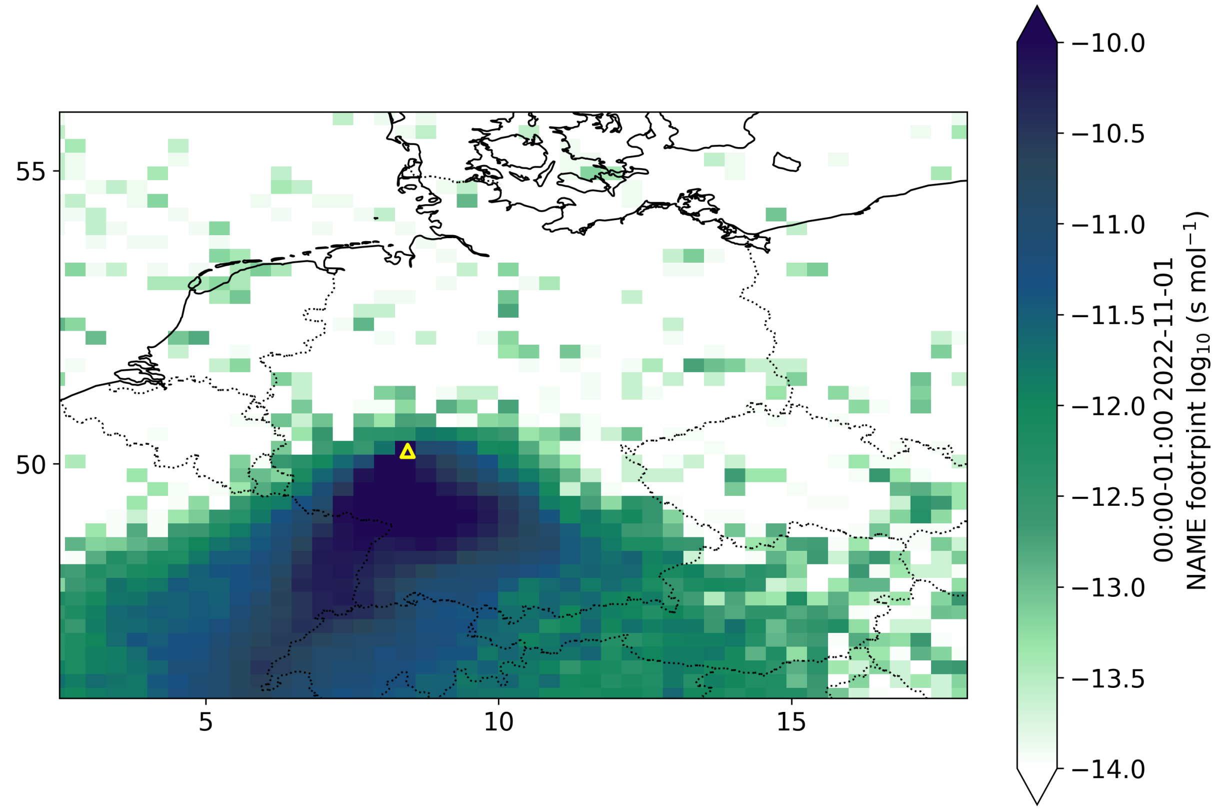

Figure 3: NAME air history on 1 November 2022 between 00:00 and 01:00 UTC at the site TOB (yellow triangle). During this period, a strong pollution event was recorded (see Figure 2 A).

To assess how sensitive each site is to the emissions from the focus region, we calculated the sensitivity of the focus region within each NAME footprint for 2023 (see Figure 4). These sensitivities were additionally normalised to the value at Taunus Observatory: Gartow = 0.15, Steinkimmen = 0.14, Lindenberg = 0.27, Taunus Observatory = 1, Ochsenkopf = 1.18, Karlsruhe = 6.02, Hohenpeissenberg = 0.37 and Zugspitze = 0.21. It should be noted that this analysis is based on averages over the whole year 2023.

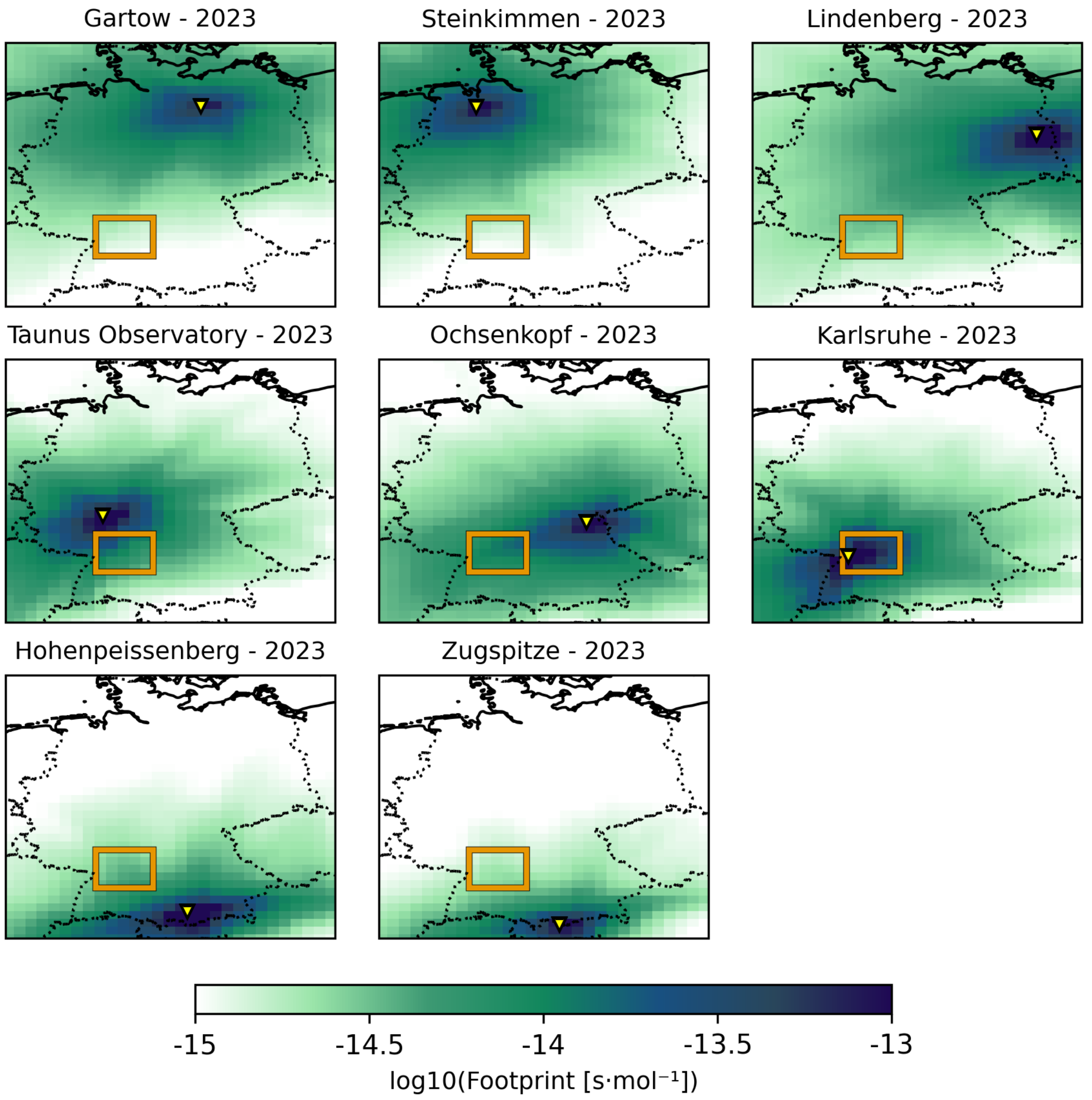

Figure 4: Averaged NAME air history in 2023 for all German sites (yellow triangle). The focus region (48.637 °N – 49.807 °N, 8.404 °E – 10.164 °E) of this study is indicated in orange.

Correlation InTEM vs. Observations

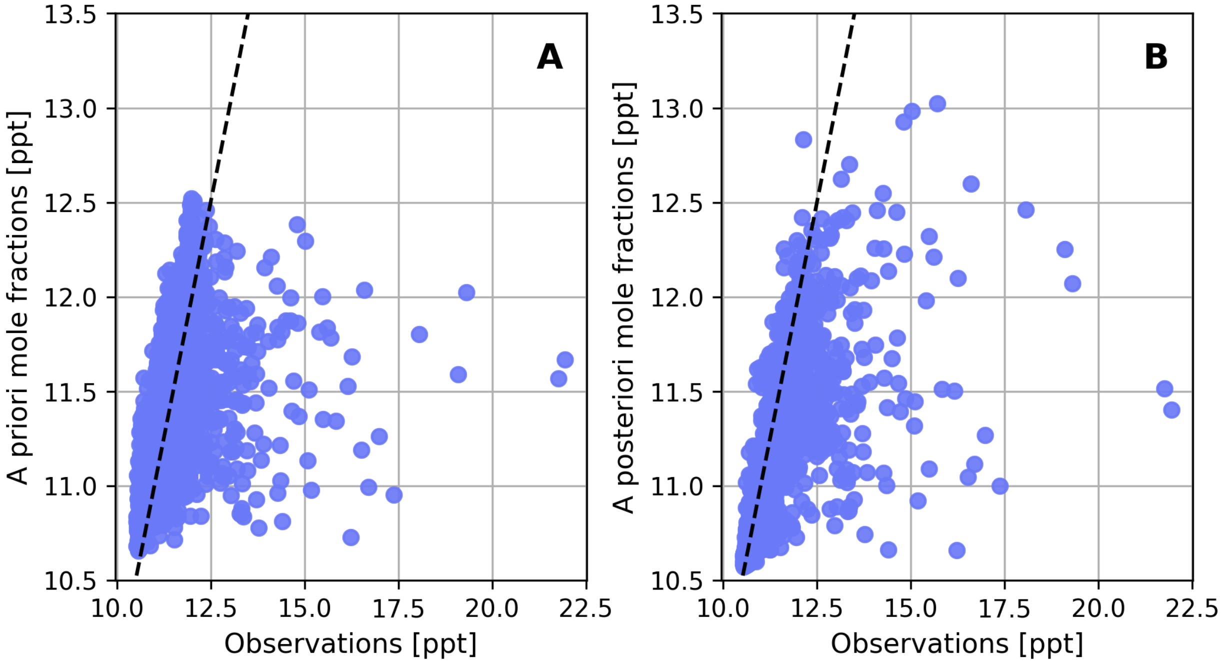

Figure 5: A: Correlation between observations and a priori mole fractions modelled at TOB (Pearson correlation coefficient: 0.48, p value < 0.01). B: Correlation between observations and a posteriori mole fractions modelled at TOB (Pearson correlation coefficient: 0.63, p value < 0.01).

Additional InTEM results

Table 3: Comparison of SF_6 emission estimates (tonnes) for Germany and the focus region (48.637 °N – 49.807 °N, 8.404 °E – 10.164 °E) of the InTEM run of the study (initial run), an InTEM run without the two sites TOB and KIT and an InTEM run without all German sites.

InTEM version		2020	2021	2022	2023
Initial run	Germany	112 ± 26	118 ± 20	93 ± 19	89 ± 15
	focus region	59 ± 16	35 ± 13	32 ± 12	29 ± 11
Without TOB and KIT	Germany	95 ± 25	118 ± 20	94 ± 20	96 ± 16
	focus region	36 ± 20	37 ± 19	38 ± 19	25 ± 15
Without German sites	Germany	88 ± 30	96 ± 28	77 ± 27	102 ± 21
	focus region	32 ± 24	47 ± 20	41 ± 18	2 ± 17

To assess how the additional German monitoring sites affect the inversion modelling, we performed two separate runs: one excluding the two most polluted sites (TOB and KIT) and one excluding all measurement sites in Germany. The corresponding emission estimates for Germany and the focus region are listed in Table 3, while the resulting emission maps are shown in Figure 6 (InTEM run without TOB and KIT) and in Figure 7 (InTEM run without German monitoring sites). Both the emission maps and the estimated emissions in Germany and in the focus region support our analysis: a clear emission hotspot is present in southwestern Germany. However, as the number of German observations decreases, the model's ability to identify the point source diminishes but does not remove: it decreases the accuracy of the location in the southwest of Germany. Furthermore, the emission estimates in the model run without the German monitoring stations are for the years 2020 - 2022 noticeably lower (e.g. in 2020: 112 ± 26 t total emissions in Germany (initial run), 88 ± 30 t total emissions in Germany (run without German sites)), but higher in 2023 compared to the initial run (89 ± 15 t total emissions in Germany (initial run), 102 ± 21 t total emissions in Germany (run without German sites)). Increasing the number of observational data integrated into the inversion improves the ability to attribute emissions to their respective source regions.

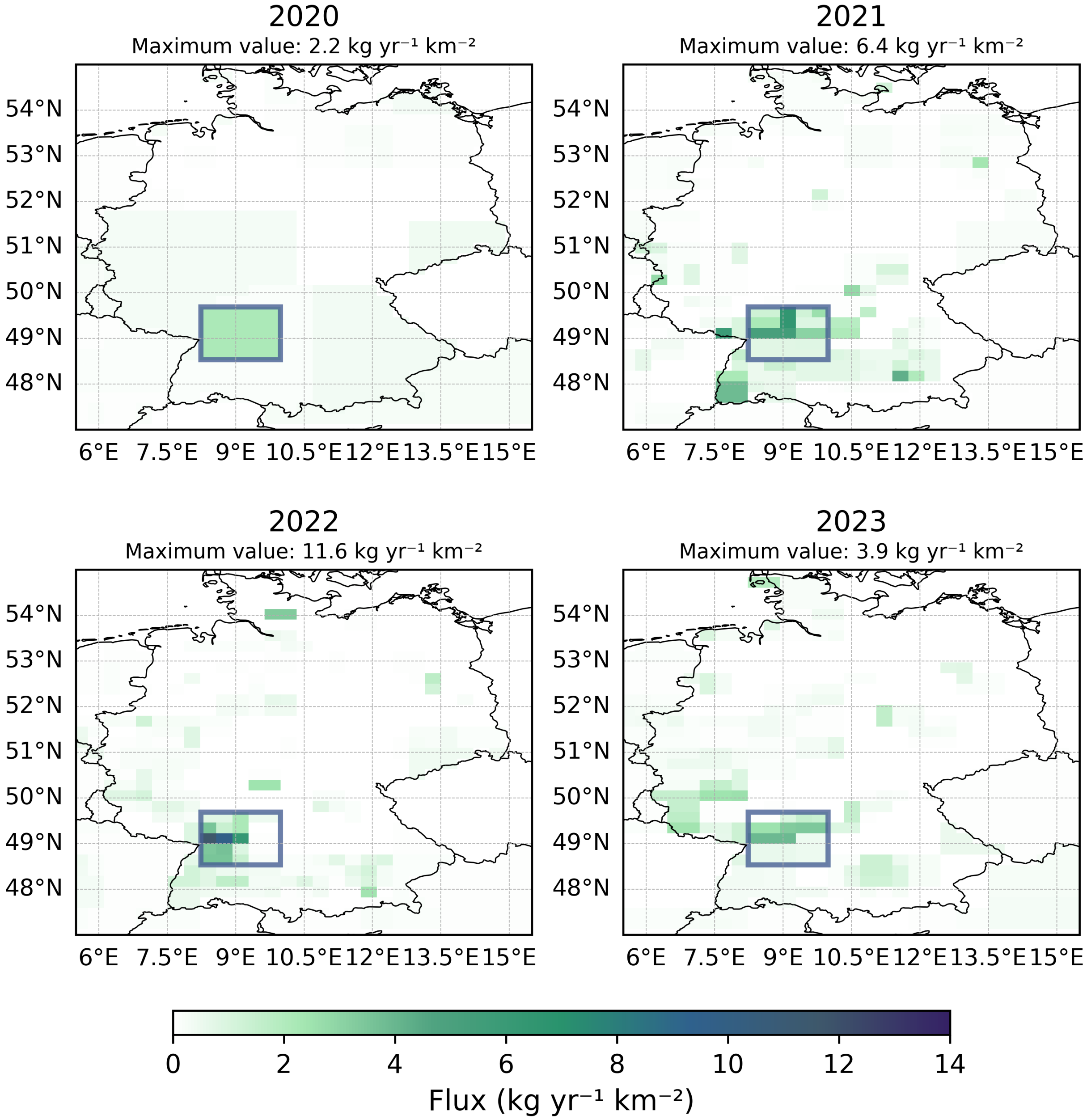

Figure 6: Annually averaged InTEM top-down inversion emission estimate for SF_6 (Flux kg $yr^{-1}km^{-2}$) without the sites KIT and TOB for the period 2020-2023 with the focus on Germany. A focus region, shown in the figures as a blue box (48.637 °N – 49.807 °N, 8.404 °E – 10.164 °E), was defined and analysed to characterise emissions in the high emission region. The highest emission value of a grid cell in each respective year is shown in the figure.

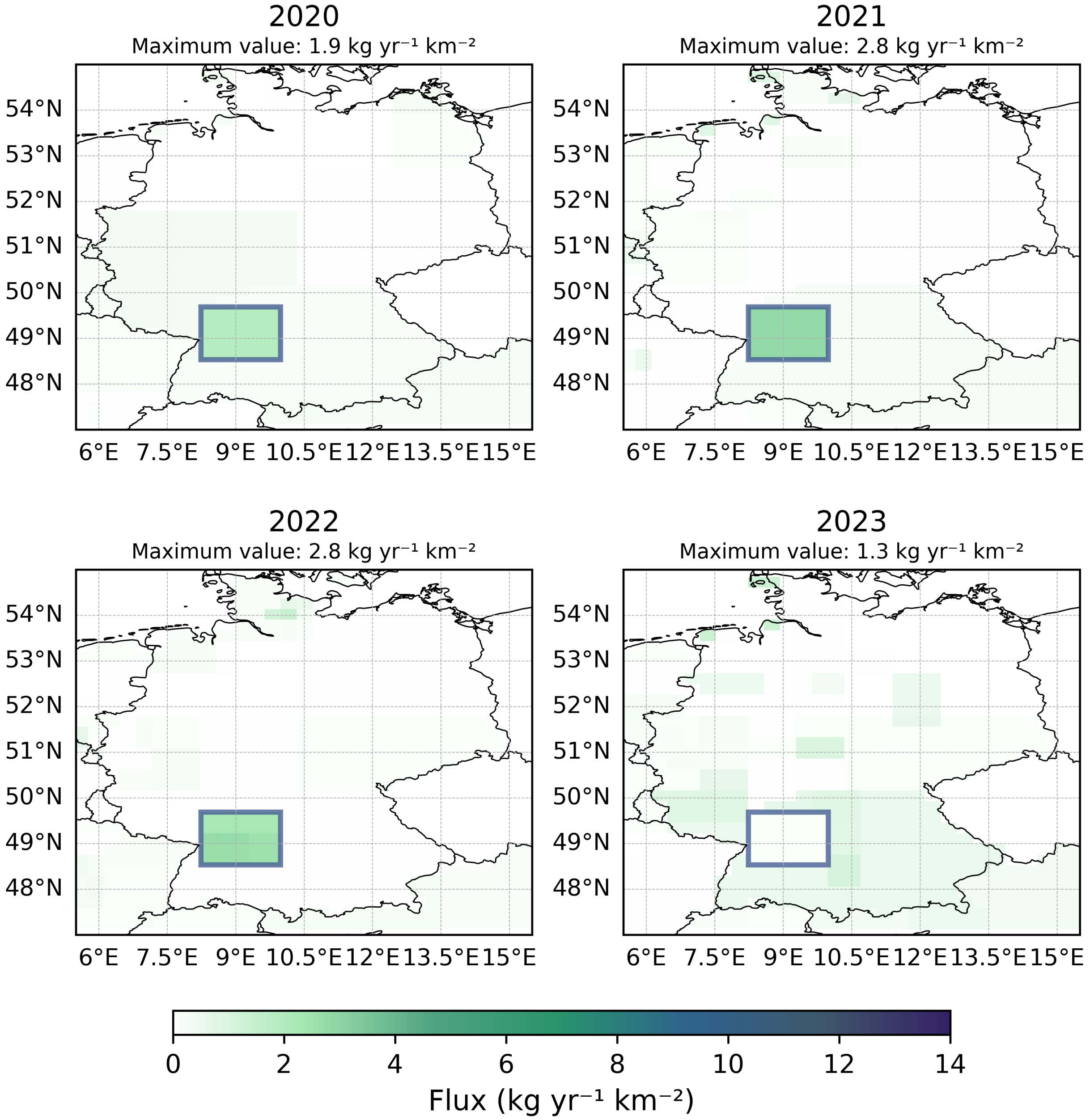

Figure 7: Annually averaged InTEM top-down inversion emission estimate for SF_6 (Flux kg $yr^{-1}km^{-2}$) without German measurement sites for the period 2020-2023 with the focus on Germany. A focus region, shown in the figures as a blue box (48.637 °N – 49.807 °N, 8.404 °E – 10.164 °E), was defined and analysed to characterise emissions in the high emission region. The highest emission value of a grid cell in each respective year is shown in the figure.

FLEXPART / Flexinvert+ inversions

We use the inversion method and the global data set described in detail in Vojta et al. (2024) and Vojta et al.(2025), to determine European SF_6 emissions at a 0.25° resolution using the Lagrangian particle dispersion model FLEXPART[4] and the Bayesian inversion framework Flexinvert+.[5] The results are derived from an ensemble of inversions, employing a Monte Carlo approach to randomly sample and combine key inversion parameters, to comprehensively quantify the uncertainties of inversion results, with 59 inversions conducted for every year.

Figure 8 presents the distribution of SF_6 emissions in Germany, averaged over the period 2020 – 2021. The inversion results reveal significant emissions in southwestern Germany. To estimate emissions of this area, we aggregate emissions inside the 1.75° x 1.75° focus region 1 with boundaries at 48.75°N – 50.0°N, 8.25°E – 10°E. It is important to note that our European inversions strongly smooth point source emissions over larger areas. While quantifying emissions in a region will also include other emissions, it may still provide a better representation of the point source emissions than quantifying the emissions in a single grid cell containing the point source. However, it also increases the possibility of capturing contributions from other potential sources within the defined area.

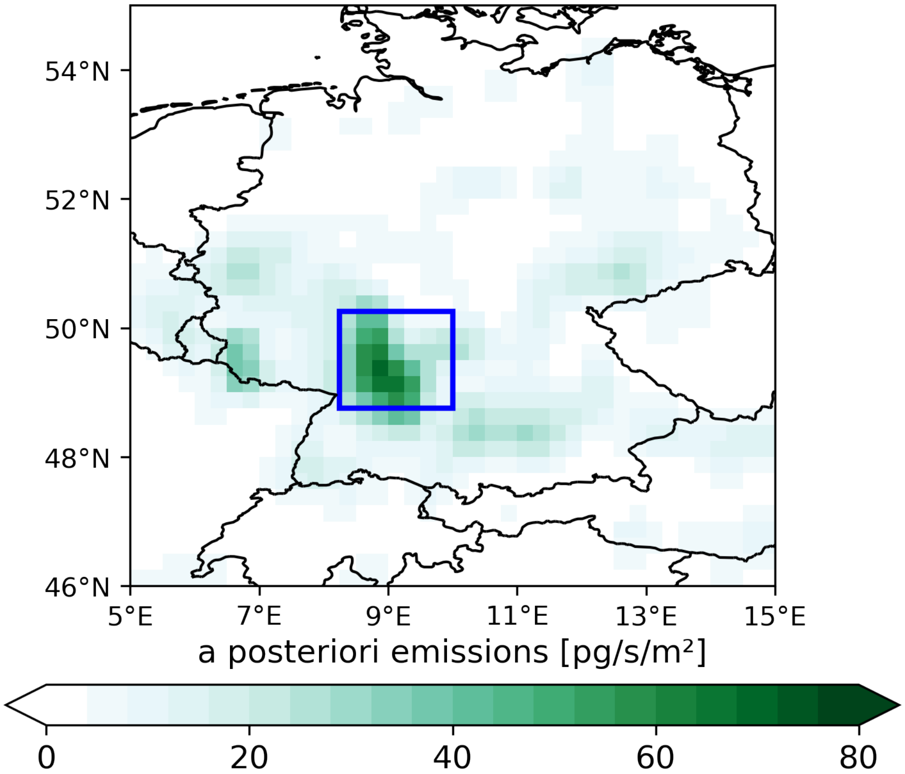

Figure 8: Averaged a posteriori emission distribution of SF_6 over Germany for the period 2020–2021, based on FLEXPART / Flexinvert+. The blue box delineates the boundaries of focus region 1, used to investigate emissions from the area of elevated fluxes.

EDGAR gridded inversion 2020 – 2023

The map in Figure 9 shows the averaged spatial distribution of SF_6 emissions over Germany (kg km^{-2}) based on EDGAR data[6] for the period 2020 – 2023. As these estimates are partly derived from bottom-up inventories, they show a different spatial pattern compared to the two top-down inversion approaches used in this study. The higher emissions around cities can be attributed to the assumed emissions from soundproof windows. Within the focus region, the average EDGAR SF_6 emissions over 2020 – 2023 amount to 9 t yr^{-1}, which is substantially lower than the averaged emissions estimated by InTEM over the same period (39 $\pm$ 13 t yr^{-1}).

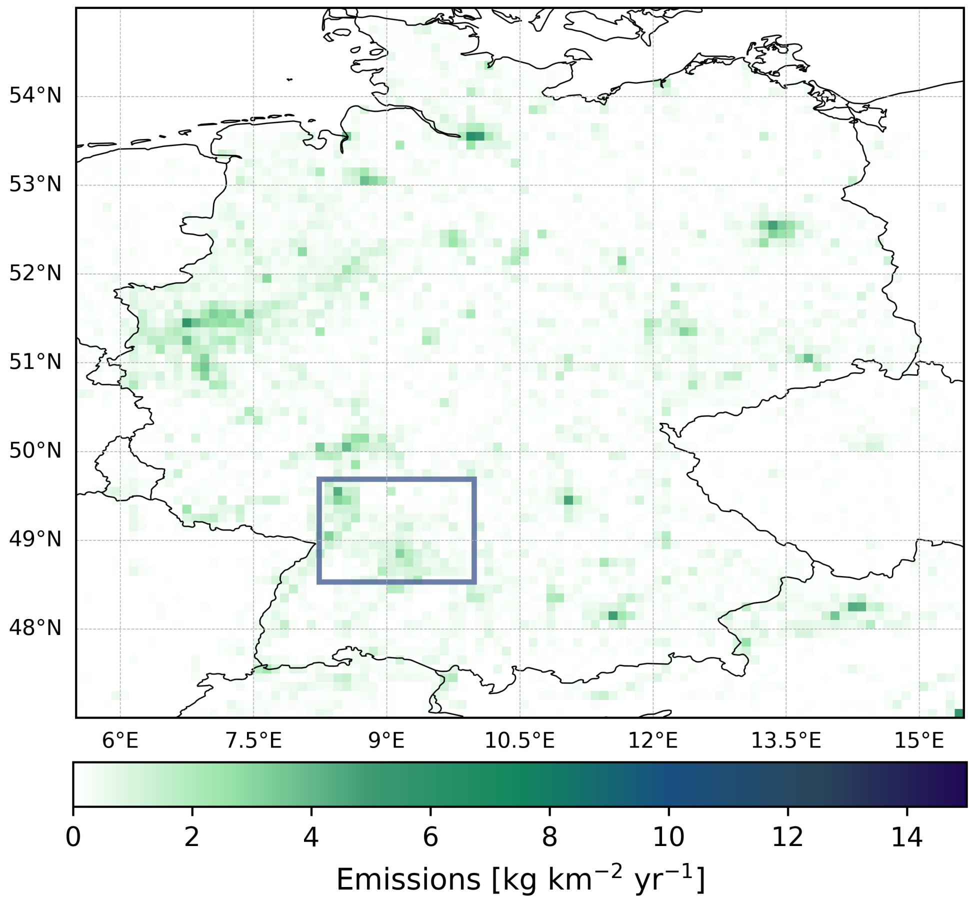

Figure 9: Averaged gridded EDGAR emission distribution of SF_6 over Germany for the period 2020–2023.[6] The blue box delineates the boundaries of focus region 1 (48.637 °N – 49.807 °N, 8.404 °E – 10.164 °E), used to investigate emissions from the area of elevated fluxes.

References

(1) Prinn, R. et al. AGAGE Data – Carbon Dioxide Information Analysis Center (CDIAC), Oak Ridge National Laboratory (ORNL), Oak Ridge, TN (United States), ESS-DIVE repository. **2018**, 10.3334/CDIAC/ATG.DB1001, last access: 2025-06-03.

(2) Vojta, M.; Plach, A.; Annadate, S.; Park, S.; Lee, G.; Purohit, P.; Lindl, F.; Lan, X.; Mühle, J.; Thompson, R. L.; Stohl, A. A global re-analysis of regionally resolved emissions and atmospheric mole fractions of SF_6 for the period 2005 – 2021. *EGUsphere* **2024**, 1–48, 10.5194/egusphere-2024-811.

(3) Vojta, M.; Plach, A.; Thompson, R. L.; Purohit, P.; Stanley, K.; O'Doherty, S.; Young, D.; Pitt, J.; Lan, X.; Stohl, A. A thousand inversions to determine European SF_6 emissions from 2005 to 2021. *EGUsphere* **2025**, 10.5194/egusphere-2025-1095.

(4) Pisso, I. et al. The Lagrangian particle dispersion model FLEXPART version 10.4. *Geoscientific Model Development 12*, 10.5194/gmd-12-4955-2019.

(5) Thompson, R. L.; Stohl, A. FLEXINVERT: an atmospheric Bayesian inversion framework for determining surface fluxes of trace species using an optimized grid. *Geoscientific Model Development* 10.5194/gmd-7-2223-2014.

(6) IEA et al. *GHG emissions of all world countries*; Publications Office of the European Union, 2024; doi/10.2760/4002897.
